# Comparison of methods for ranking and stratification of community-dwelling adults by pericardial fat thickness measured using cardiovascular magnetic resonance imaging

**DOI:** 10.1186/1532-429X-11-S1-P203

**Published:** 2009-01-28

**Authors:** Michael L Chuang, Noriko Oyama, Philimon Gona, Carol J Salton, Rahul R Jhaveri, Daniel Levy, Christopher J O'Donnell, Warren J Manning

**Affiliations:** 1 Beth Israel Deaconess Medical Center, Boston, MA USA; 2 The NHLBI's Framingham Heart Study, Framingham, MA USA

**Keywords:** Right Ventricular, Cardiovascular Magnetic Resonance, Pericardial Effusion, Cardiovascular Magnetic Resonance Imaging, Right Ventricular Free Wall

## Introduction

Pericardial adipose tissue is associated with excess cardiovascular risk factors and may itself be a marker for and modulator of cardiovascular disease, possibly through paracrine activity as pericardial adipose tissue is metabolically highly active. In many research studies pericardial fat thickness (PFT) is measured using transthoracic echocardiography from the parasternal long-axis view, from which PFT over the right ventricular (RV) free wall is determined. However, the ability of echocardiography to measure PFT is limited by acoustic window issues and potential confounding with pericardial effusion. Pericardial fat is readily seen on routine CMR imaging for ventricular function, but there is no standard approach to assessment of pericardial adipose burden by CMR. For simplicity and to facilitate comparison with the literature, we elected to measure RV free wall PFT by CMR from the 4-chamber view. In this abstract we sought to determine whether measurement of PFT at its maximum versus at a predefined location (mid-RV level; 4ChMID) would change stratification of subjects by PFT.

## Methods

500 adults (aged 61 ± 9 years, 250 women and 250 men) were randomly selected from the Framingham Offspring Cohort CMR study database. Subjects were imaged on a 1.5-T scanner (Philips Medical Systems) using a cardiac array coil for RF signal reception. PFT was measured at end-diastole from the 4-chamber view using a cine SSFP sequence (TR/TE/θ = 3.3/1.7/60°, 10-mm slice thickness, in-plane resolution 1.92 × 1.56 mm^2^) over the mid-level right ventricle (4chMID) and at maximal thickness in the same orientation (4chMAX). Sex-specific ranking of subjects by PFT was performed for 4chMID and for 4chMAX. Changes between 4chMID and 4chMAX ranks were summarized as median [interquartile] differences. Additionally, subjects were then stratified by quintiles of PFT. We assessed concordance of individual subject rankings by Spearman correlation (r_s_) and tabulated number of across-quintile changes for each sex.

## Results

**Figure 1:**
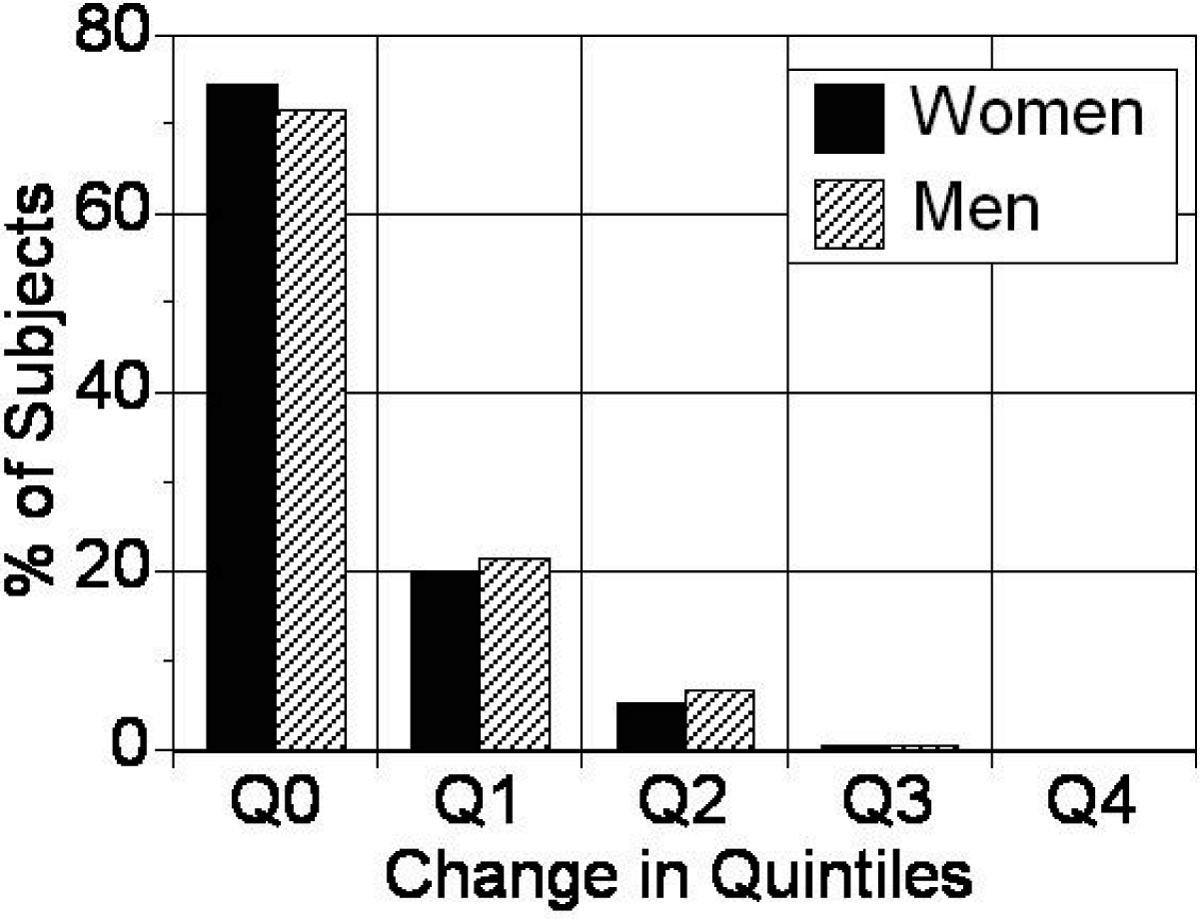
Figure 1

PFT could be measured successfully for all subjects, with good reproduciblity between readers (interclass correlation coefficients: interobserver ICC = 0.89, intraobserver ICC = 0.98). Men had significantly greater PFT than women for both 4chMAX (17.8 ± 8.0 vs. 14.2 ± 6.1 mm, p < 0.0001) and 4chMID (10.7 ± 7.2 vs. 8.3 ± 5.1 mm, p < 0.0001). There was good correlation for ranking of PFT by 4chMAX and 4chMID measurements for both men (r_s_ = 0.754) and women (r_s_ = 0.763), both p < 0.0001. The median [interquartile] rank-differences were 31 [14, 56] for men and 30 [15, 50] for women. Figure 1 shows percentage of subjects whose quartile-ranking remained the same (Q0) or changed by 1 quintile (Q1), or 2, 3, or 4 quintiles (Q2, Q3 and Q4 respectively).

## Conclusion

Men have greater PFT than women, but concordance in ranking between 4ChMAX and 4ChMID PFT was similar for both sexes. As ranking of subjects was similar regardless of whether maximal or mid-RV PFT was used, it may be reasonable to select a standardized measurement method, i.e. mid-RV level PFT, for consistency. Further work is needed to determine whether PFT measured by CMR has additional prognostic value over standard clinical predictors of cardiovascular risk, but PFT can be determined reliably and reproducibly using routine SSFP sequences for assessment of ventricular function, so that PFT can be determined without increasing examination time in a standard clinical CMR examination.

